# Early-life oxytocin attenuates the social deficits induced by caesarean-section delivery in the mouse

**DOI:** 10.1038/s41386-021-01040-3

**Published:** 2021-05-26

**Authors:** Livia H. Morais, Anna V. Golubeva, Sophie Casey, Karen A. Scott, Ana Paula Ramos Costa, Gerard M. Moloney, Timothy G. Dinan, John F. Cryan

**Affiliations:** 1 APC Microbiome Ireland, University College Cork, Cork, Ireland; 2 Department of Anatomy and Neuroscience, University College Cork, Cork, Ireland; 3 Department of Psychiatry and Neurobehavioural Science, University College Cork, Cork, Ireland; 4 Departamento de Farmacologia, CCB, UFSC, Florianópolis, Brazil; 5 Present Address: Division of Biology and Biological Engineering, California Institute of Technology, Pasadena, CA USA; 6 Present Address: Irish Centre for Foetal and Neonatal Translational Research, INFANT, Cork, Ireland; 7 Present Address: Department of Pharmacodynamics, McKnight Brain Institute, College of Pharmacy, University of Florida, Gainesville, FL USA

**Keywords:** Emotion, Pharmacology

## Abstract

The oxytocin (OXT) system has been strongly implicated in the regulation of social behaviour and anxiety, potentially contributing to the aetiology of a wide range of neuropathologies. Birth by Caesarean-section (C-section) results in alterations in microbiota diversity in early-life, alterations in brain development and has recently been associated with long-term social and anxiety-like behaviour deficits. In this study, we assessed whether OXT intervention in the early postnatal period could reverse C-section-mediated effects on behaviour, and physiology in early life and adulthood. Following C-section or *per vaginum* birth, pups were administered with OXT (0.2 or 2 μg/20 μl; s.c.) or saline daily from postnatal days 1–5. We demonstrate that early postnatal OXT treatment has long-lasting effects reversing many of the effects of C-section on mouse behaviour and physiology. In early-life, high-dose OXT administration attenuated C-section-mediated maternal attachment impairments. In adulthood, low-dose OXT restored social memory deficits, some aspects of anxiety-like behaviour, and improved gastrointestinal transit. Furthermore, as a consequence of OXT intervention in early life, OXT plasma levels were increased in adulthood, and dysregulation of the immune response in C-section animals was attenuated by both doses of OXT treatment. These findings indicate that there is an early developmental window sensitive to manipulations of the OXT system that can prevent lifelong behavioural and physiological impairments associated with mode of birth.

## Introduction

Birth delivery by Caesarean-section (C-section), when medically indicated, is a crucial life-saving procedure. However, in recent decades, the use of elective C-section has dramatically increased worldwide, with rates exceeding World Health Organisation guidelines (of 10–15%), especially in middle- and high-income countries [1]. This trend raises significant concerns given the growing evidence for an association between C-section delivery and increased risk for immune and metabolic disorders [2–5]. Birth by C-section has been linked to possible neurodevelopmental consequences in humans [6–8] and in animals [9–11]. Recently, we have demonstrated that mice delivered by C-section exhibit enduring behavioural deficits [9].

OXT is a key modulator of mammalian maternal-offspring attachment and social behaviour [12–16]. The OXT system can be activated by stressful and anxiogenic stimuli and acts as key-modulator of the hypothalamic-pituitary-adrenal (HPA) axis [17–19]. Consistent with the important role of OXT in social behaviour promotion and stress regulation, OXT dysregulation has been associated with anxiety and autism spectrum disorders (ASD) [20–23]. Therefore, there is a growing interest in the OXT system as a potential target in the treatment of neuropsychiatric disorders associated with stress and social dysfunction [24–29].

In addition to the widely studied effects of OXT on behaviour, there is increasing evidence that the OXT system plays an important role in integrating neural, endocrine, metabolic, and immune information that is crucial for development and function of the immune system. Thus OXT can act indirectly on immunity; as it attenuates plasma tumour necrosis factor alpha (TNF-α), interleukin (IL)−1, IL-4, IL-6 and other inflammatory mediators in parallel with attenuating stress-related hormones, such as cortisol in healthy individuals challenged with endotoxin [30]. OXT can also directly bind to OXTR and vasopressin receptors (AVPR) expressed in major components of the immune system including thymic cells [31].

Behavioural and physiological adaptative effects of OXT is thought to start at birth [32]. Indeed early life OXT prevents respiratory distress and can prevent birth-related anoxia [33], participates in gamma aminobutyric acid (GABA) system maturation [34–36], on fetal analgesia [37] and triggers catecholamine and cytokine release [38]. Furthermore, the OXT system starts to develop *in utero* and the peak of OXT receptor (OXTR) binding occurs prior to weaning and coincides with critical periods for brain wiring and development [39]. This overlapping time-window leads to increased vulnerability of the OXTR to adverse early-life experiences [40]. In this context, early-life pharmacological manipulations of the OXT system, and their subsequent impact on behaviour and physiological changes are of great interest. These studies are relevant in the context of human neurodevelopmental disorders, as there are growing numbers of human studies investigating the efficacy of OXT infusions for ameliorating symptoms in individuals with ASD [23, 41, 42]. Perinatal administration of synthetic OXT (pitocin) is a common obstetric practice to accelerate childbirth, and OXTR antagonists are often administered in order to prevent premature labour [43]. Interestingly, OXT administration during labour has been associated with higher odds of C-section [44] with consequences that remain be fully-explored. Given the multitude of effects that OXT can have on health and neurodevelopment, it is important to better characterise the effects of these manipulations. Despite that, it remains unclear whether interventions targeting the OXT system can ameliorate the enduring effects of C-section on physiology and behaviour. Therefore, in this study, we investigated the long-term effects of early postnatal OXT treatment on mouse offspring delivered by C-section.

## Materials and methods

### Animals

The experiments were carried out in male Swiss mice of different ages. 8-week-old female and male breeders were obtained from Harlan laboratories, UK. Breeding began after 1–2 weeks of acclimatisation to the animal holding room. Animals were kept under a strict 12:12 h dark-light cycle and temperature (20 ± 1 °C, 55.5%) with food and water given ad libitum unless specified otherwise. Male offspring were weaned at postnatal (P) day 21 and group-housed in 3–4 mice per cage. Each experimental group included the offspring from 3 to 4 independent litters. In addition, 10-week-old Swiss male mice, purchased from Harlan laboratories, UK, were used as conspecifics in the three-chamber test. All experimental procedures were conducted in accordance with the Directive 2010/63/EU and were approved by the Animal Experimentation Ethics Committee of University College Cork # 2012/036.

### Mode of delivery: experimental groups

Mice were time-mated, and the presence of a vaginal plug in females was marked as a gestational day 0.5 (G0.5). Males were removed from the cages, and pregnant females were not disturbed except for cage cleaning. Pregnant dams were randomly assigned to one of two experimental groups: (1) Vaginal delivery: the offspring were delivered naturally and raised by their biological mother. (2) C-section: the offspring were delivered by C-section surgery and raised by a foster dam that gave birth on the same day. Treatments were assigned to the entire litter; each pup in a litter received the same treatment (See **OXT administration** for further details). After weaning, mice were group-housed in cages accordingly to the litter.

### C-section surgery

At full term (G19.5), female mice were euthanized by cervical dislocation. All further procedures were performed in clean conditions. The abdominal skin was prepped with 70% ethanol; the uterus was excised with sterile surgical tools and placed on sterile gauze, preheated to +37 °C with a heating pad. Pups were removed from the uterus by applying a gentle pressure with a sterile swab, and further massaged for 1–2 min to clear the amniotic membrane and stimulate pulmonary breathing. Once spontaneous breathing was noted, pups were immediately transferred to a foster mother. The procedure took ~3 min. The average litter size was 10 pups/litter.

### OXT administration

From P1 to P5, male and female pups were temporarily removed from their nest (5 min), weighed and given a daily subcutaneous (s.c.) injection of OXT. Pups received 0.2 µg or 2 µg of OXT (Tocris, Bioscience, UK) dissolved in 20 µL of isotonic saline, or 20 µL of isotonic saline alone (control). Treatment was adapted from [45, 46].

### Experimental design

**Fig. 1:**
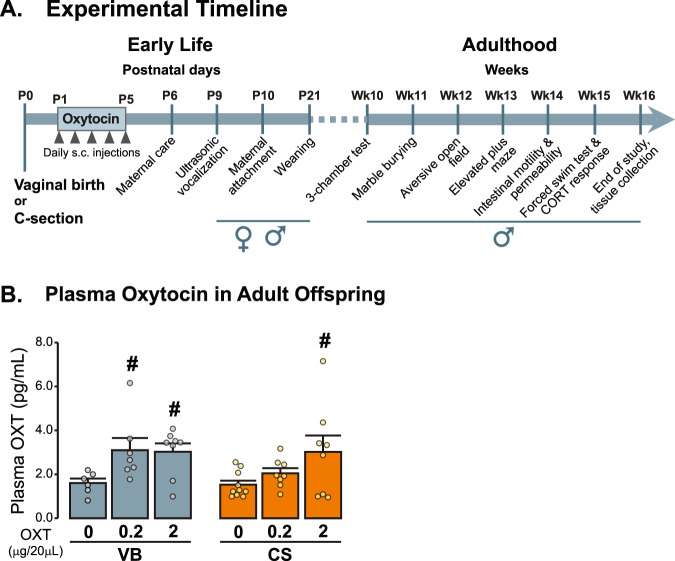
Early-life oxytocin increases plasma oxytocin concentration in adulthood. **A***Schematic representation of OXT treatment and behavioural testing sequence.* Following birth by C-Section or per vaginum, pups received daily injections of OXT (0.2 or 2 μg/20 μl saline; s.c.) from postnatal days 1–5 and were subjected to a sequence of behavioural tests. **B** *OXT administration in early-life increases OXT plasma levels in adulthood.* Mode of delivery effect (*F* (1, 41) = 1.116, *p* = 0.297); treatment effect (*F* (1, 41) = 5.893, *p* = 0.006); mode of delivery × treatment (*F* (2,41) = 0.886, *p* = 0.420). ([VB control *n* = 6, VB 0.2 OT *n* = 7, VB 2 OT control *n* = 8, CS control *n* = 10, CS 0.2 OXT *n* = 8, CS 2 OXT *n* = 8]). Two-way ANOVA, followed by LSD post-hoc. OXT oxytocin, VB vaginal birth, CS C-section. Male offspring in each group derived from three independent litters.

Effects of C-section on behaviour were evaluated in both male and female offspring in early-life (P9/P10), and only in males in adulthood (weeks 10–16). The behavioural experiments were performed during the light phase and between the hours of 9 am and 2 pm. Mice were habituated to the room 30 min prior to each test. All the behavioural tests were done with the same animals. The exact order of behavioural tests, as well as resting intervals between them were optimised to reduce cumulative stress effects and the potential confounding carryover effects from previous tests (see Fig. 1a).

### Plasma OXT measurement

Trunk blood was collected and centrifuged (5 min, 5000 *g*, 4 °C), and plasma was extracted, and stored at −20 °C until OXT concentration was measured using a radioimmunoassay (RIA) performed by RIAgnosis (Sinzing; Germany).

### Gene expression analysis with qRT-PCR

Total RNA was extracted from the paraventricular nucleus of the hypothalamus (PVN) using the mirVana^™^ miRNA Isolation Kit (Thermo Fisher Scientific). RNA concentration was quantified using the ND-1000 spectrophotometer (NanoDrop^®^). Equal amounts of RNA were reverse transcribed to cDNA using a High Capacity cDNA Reverse Transcription Kit (Applied Biosystems, Life Technologies, Carlsbad, CA). Gene expression was analysed using TaqMan Gene Expression Assays on the AB7300 system (Applied Biosystems, Thermo Fisher Scientific). Changes in gene expression levels were calculated using the ΔΔCt method [47]. The average of three technical replicates for each biological sample was normalised to β-actin (*Actb*) expression, then the fold changes vs. the control (VB) group were calculated. The expression of *Actb* was stable across all experimental groups.

### Behavioural assessments

#### Maternal care

Maternal care was assessed at P6 in order to exclude possible cross-fostering confounding effects. Home cages were transported to the experimental room 30 min prior to the experiment. The behaviour of each dam was monitored for 30 min in the morning in their home cage. The time spent on nursing, pup licking/grooming, carrying and time on-nest were collectively considered as high-care behaviours. The data are presented as time engaged in high care behaviours.

#### Maternal attachment and social recognition behaviour (homing test)

The homing test evaluates the tendency capacity of pups to recognise their mother’s nest and is an indirect measure of attachment and social behaviour [48]. At P10, a clean mouse cage was divided into the three equallysized quadrants by wire-mesh dividers. One of the side quadrants (Maternalf) was uniformly covered with wood shavings from the home cage, thus containing familiar odour stimuli. The opposite side quadrant (Other) was covered with wood shavings from the cage of another litter (born at approximately the same time); the middle quadrant (Neutral) was covered with clean bedding material. Male and female pups were placed individually in the middle quadrant for 1 min; the dividers were then removed, and the pups allowed to move freely around for 2 min. Total time spent in each area was recorded. Percentage maternal preference was calculated using the formula: (time spent in the mother’s bedding (s)*100/duration of the test (120 s)).

#### Ultrasonic vocalisation test (USV)

The USV was performed as described [9]. Male and female pups were isolated and placed into a clean plastic container enclosed in a sound-attenuating chamber for 3 min. USV calls were detected by an ultrasound sensitive microphone—a bat detector (US Mini-2 bat detector, Summit, Birmingham, USA) tuned in the range of 60–80 kHz and the total number of calls was recorded using the software Noldus UltraVox version 2.0 (Tracksys, UK).

#### Three-chamber test

Sociability and preference for social novelty were assessed in a three-chamber apparatus, as described previously [49]. Animals were placed in a rectangular apparatus divided into three chambers separated by transparent partitions with small circular openings allowing easy access to all compartments. The test was composed of three sequential 10 min trials: (1) habituation (a test animal was allowed to explore the three empty chambers); (2) sociability (an unfamiliar animal was positioned in a wire-mesh cage in either the left- or right- side chamber); (3) social novelty preference (a novel animal was positioned in either right- or left- side chamber, opposite the one occupied by the now familiar animal). The time of active interaction was measured. All animals were age- and sex-matched.

#### Marble burying test

The marble burying test measures repetitive and anxiety-like behaviours; the number of marbles buried during the test correlates with anxiety [50]. Clean cages were filled with a 4 cm layer of chipped cedar wood bedding. Twenty glass marbles (15 mm diameter) were laid on top of the bedding, equidistant from each other in a 4 × 5 arrangement. The mouse was then placed in the cage and allowed to explore it for 30 min. The number of marbles buried (>2/3 marble covered by bedding material) was noted.

#### Elevated plus maze test (EPM)

The elevated plus maze consists in a Plexiglas plus-shaped apparatus with two open and two enclosed arms (50 cm × 5 cm × 15 cm walls) elevated from the floor by 1 m. Mice were placed in the centre of the EPM apparatus facing an open arm and allowed to explore it for 5 min. The number of entries into open and closed arms were noted. The experiment was performed in the dark under red light. Mice were habituated to the room and light conditions for 1 h prior to the start of behavioural test.

#### Aversive open field test (OF)

The aversive open field test is used to assess locomotor activity, as well as the response to a novel, stressful environment [51]. Light was set at 1000 lux. The distance moved in an open-field arena (Perspex box with white base: 30 × 30 × 20 cm) and the time spent in the central zone (16 × 15 cm) of the open field were recorded during a 10 min period using Ethovision videotracking system (Noldus Information Technology).

#### Forced-swim test (FST)

Mice were placed into a cylinder filled with tepid water (23–25 °C) to a depth of 17 cm. Behaviour was recorded by a camera positioned from above the swim tank. The immobility time was scored during the last 4 min of the 6 min test. After removal from the cylinder, mice were placed into a separate cage for recovery.

#### Analysis of cytokine release in stimulated murine splenocyte

Splenocytes were isolated as follows: immediately upon collection, cellular contents of spleen were trimmed of all fat and dissociated in RPMI medium (R8758, supplemented with 10% FBS and 5% penicillin/streptomycin, Sigma), treated with Red Blood Cell lysis buffer (Sigma) to remove erythrocytes, passed through a 70 μm filter, and resuspended in fresh RPMI medium (as above) for seeding. Splenocytes were seeded in 6 well plates at 16 × 10^6^ cells/well in 4 mL. Following a 2.5 h rest period, cells were stimulated with lipopolysaccharide (LPS-2 μg/mL) for 24 h. Following stimulation, the supernatants were harvested to assess IL-4, IL-6, IL-10 and TNF-α concentration (pg/mL) using the Proinflammatory Panel 1 (mouse) V-PLEX MULTI-SPOT^®^ Meso Scale Discovery kits (MSD, Rockville, MD, USA) as per manufacturer’s instructions.

#### Gastrointestinal transit

Intestinal transit time was measured as previously described [52]. Mice were single-housed and habituated to new cages for 3 h. Following the acclimatisation, mice received a 200 µL oral gavage of carmine (C1022; Sigma Aldrich) suspended in 0.5% carboximethylcelulose sodium salt (Sigma; St Louis, MO, USA). Cages were checked every 10 min, and the time between the gavage and the appearance of the first red-coloured faecal bolus was noted.

#### In vivo intestinal permeability assay

Mice were fasted overnight and Fluorescein Isothiocyanate (FITC)-dextran, MW = 4 kDa (FD4, Sigma) was given orally by gavage, (600 mg/kg, 80 mg/ml) in phosphate buffered saline (PBS). A blood sample (100 μl in heparin-coated glass capillary) was taken from tail vein 2 h after oral gavage. Samples were kept on ice, centrifuged at ~3500 *g* for 15 min, plasma was aspirated. Plasma FITC concentrations were measured with a Victor spectrometer. The excitation maximum is 490 nm, the emission maximum was 520 nm (measured at 535 nm). Serial dilutions of FITC in PBS were prepared for standard curve.

#### Statistical analyses

Statistical analyses were carried out using SPSS version 19 (Armonk, NY, USA). Parametric data were analysed by Two-way ANOVA for mode of delivery and treatment factors followed by LSD post-hoc tests or Paired Student *t* test and represented as mean ± S.E.M. Non-parametric data were analysed by Kruskal–Wallis followed by U-Mann–Whitney tests and One-Sample Chi-Square Test. Non-parametric data are represented as median with interquartile range; whiskers represent min and max values. *p* < 0.05 was considered statistically significant. A mixed-effects regression model was used to re-analyse our data using litter as a fixed effect in order to examine the covariance structure that is inherent in the experimental design using R (version 4.0.4). *p* value < 0.05 was deemed significant; *F* and *p* values are presented in the Supplementary Table 1.

## Results

### Early-life OXT increases plasma OXT concentration in adulthood

Following C-section or *per vaginum* birth, OXT or saline was administered daily to pups from postnatal day 1–5. Social and anxiety-like behaviour and subsequent physiological and molecular parameters were assessed. To determine the long-term impact of early-postnatal treatment with OXT on the OXT system, we measured the concentration of OXT in the plasma of VB and CS adult mice. Although, there was no significant effect of the mode of delivery, a sustained increase in the hormone concentration was observed in VB and CS mice following high-dose OXT administration in early in life (Fig. 1b). However, only the VB group showed elevated plasma OXT levels in response to low-dose OXT (Fig. 1b). The synthesis and release of OXT is regulated by the activation of OXTR and AVPR in the paraventricular nuclei and in the supraoptic nuclei of the hypothalamus [53]. In addition, C-section has been shown to cause a long-term change in the PVN number of vasopressin neurons [10]. The PVN is also a major site of regulation of stress [54]. Therefore, we hypothesised that *Oxtr* and *AVP1a* receptor mRNA expression in this region would provide us with evidence of how the exposure to a stressful stimulus at birth might regulate the OXT system. However, no differences were detected between the groups (Fig. S1).

### Postnatal OXT reversed C-section mediated social recognition deficits in early-life and in adulthood

**Fig. 2:**
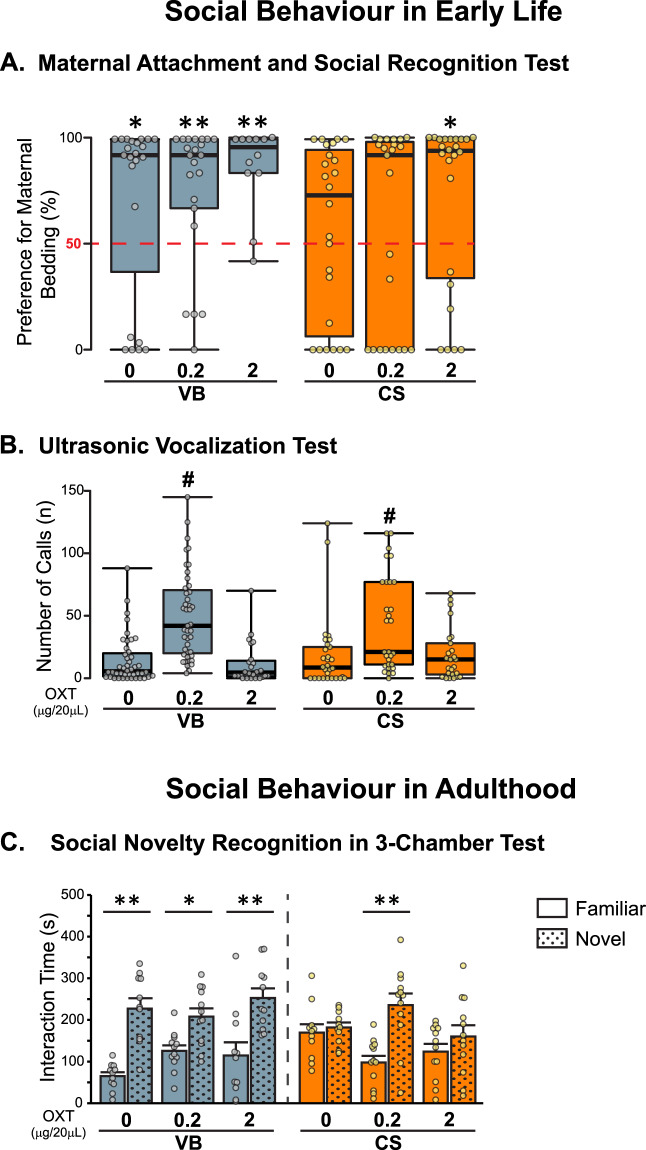
Early postnatal administration of OXT rescued C-section mediated effects on social behaviour. **A***High-dose OXT administration reversed C-section mediated effects on attachment behaviour in early-life.* VB control *x*^2^(1) = 5.261, *p* = 0.022, *n* = 23; VB 0.2 OXT *x*^2^(1) = 8.909, *p* = 0.003, *n* = 22; VB 2 OXT *x*^2^(1) = 8.333, *p* = 0.004, *n* = 12; CS control *x*^2^(1) = 0.667, *p* = 0.414, *n* = 24; CS 0.2 OXT *x*^2^(1) = 0.391, *p* = 0.532, *n* = 23; CS 2 OXT *x*^2^(1) = 4.167, *p* = 0.041, *n* = 24 (one sample Chi-square test). Data are represented as median with interquartile range; whiskers represent min and max values. Male and Female offspring. *p < 0.05 for differences within the group. **B** *Low-dose OXT increases the number of calls followed isolation-induced USV.* There was no significant effect of the mode of delivery (*x*^2^ = 0.827; df = 1; *p* = 0.363). However, when pups were treated with low-dose OXT there was a significant increase on the number of calls emitted in both birth conditions (*x*^2^ = 44.995; df = 2; *p* < 0.001). ([VB control *n* = 44, VB 0.2 OT *n* = 43, VB 2 OT control *n* = 24, CS control *n* = 30, CS 0.2 OXT *n* = 29, CS 2 OXT *n* = 25]). Male and Female offspring. Kruskal–Wallis test followed by Mann–Whitney U test. Data are represented as median with interquartile range; whiskers represent min and max values. ^#^*p* < 0.05 for treatment effect within the group. **C** *Low-dose OXT rescued the deficit in the preference for social novelty in adult CS mice.* VB control *t* (11) = 4.681, *p* = 0.001, *n* = 12; VB 0.2 OXT *t* (11) = 3.064, *p* = 0.011, *n* = 12; VB 2 OXT *t* (10) = 3.404, *p* = 0.007, *n* = 11; CS control *t* (11) = 0.594, *p* = 0.564, *n* = 12; CS 0.2 OXT *t* (11) = 3.671, *p* = 0.004, *n* = 12; CS 2 OXT *t* (10) = 0.970, *p* = 0.355, *n* = 12. Male offspring. Paired Student’s *t* test comparing interaction time with a familiar mouse to a novel mouse. Data are represented as Mean ± Standard Error of the Mean (S.E.M.). *p < 0.05 and **p < 0.01 for mouse vs. object (**A**–**C**) Offspring in each group is derived from three independent litters. OXT oxytocin, VB vaginal birth, CS C-section.

Birth by C-section was recently shown to have a negative impact on the ability of pups to recognise social behavioural cues and affect offspring-maternal attachment [9]. To test whether the postnatal treatment with OXT could ameliorate these effects, males and female pup’s maternal attachment behaviour was tested at P10 (Fig. 2a). As expected, VB pups from all treatment groups could discriminate between the familiar and non-familiar stimuli and move towards the mother’s nest (Fig. 2a), whereas CS offspring failed to discriminate the maternal stimuli (Fig. 2a). Strikingly, the postnatal treatment with the higher dose of OXT re-established the time spent in the mother’s bedding. To exclude the possibility that the observed differences in offspring phenotype were due to the impact of cross-fostering on nurturing per se, we assessed maternal care behaviour at P6 (Fig. S2). Noteworthy, the cross-fostering procedure did not impair maternal care behaviour towards CS offspring (Fig. S2). Interestingly, when female and male pups were treated with both doses of OXT there was a significant increase in the time the mother spent engaged in high-care behaviours (licking, grooming and arched back nursing the pups) in both VB and CS groups (Fig. S2). In early-life, communication behaviour was assessed in the isolation-induced USV test in female and male pups. (Fig. 2b). Although there was no significant effect of the mode of delivery in this test which has been shown to be more susceptible to litter effects [9], the treatment with low-dose OXT significantly increased the number of calls in both VB and CS groups (Fig. 2b).

To investigate if OXT could attenuate the enduring social recognition behaviour deficits induced by C-section, we performed the 3-chamber test in adulthood (Fig. 2c). Previous findings did not show deficits in sociability associated with the C-section delivery mode [9]. Here, we confirmed these findings and demonstrated that OXT treatment does not interfere with sociability in the adult male offspring (Fig. S3). However, when CS male mice were given the choice to interact with a novel or with a familiar mouse, they exhibited decreased preference for social novelty (Fig. 2c) which was completely restored by treatment with low-dose OXT early in life (Fig. 2c).

### Early-life OXT selectively improved but did not completely reverse the anxiety-like phenotype induced by C-section

**Fig. 3:**
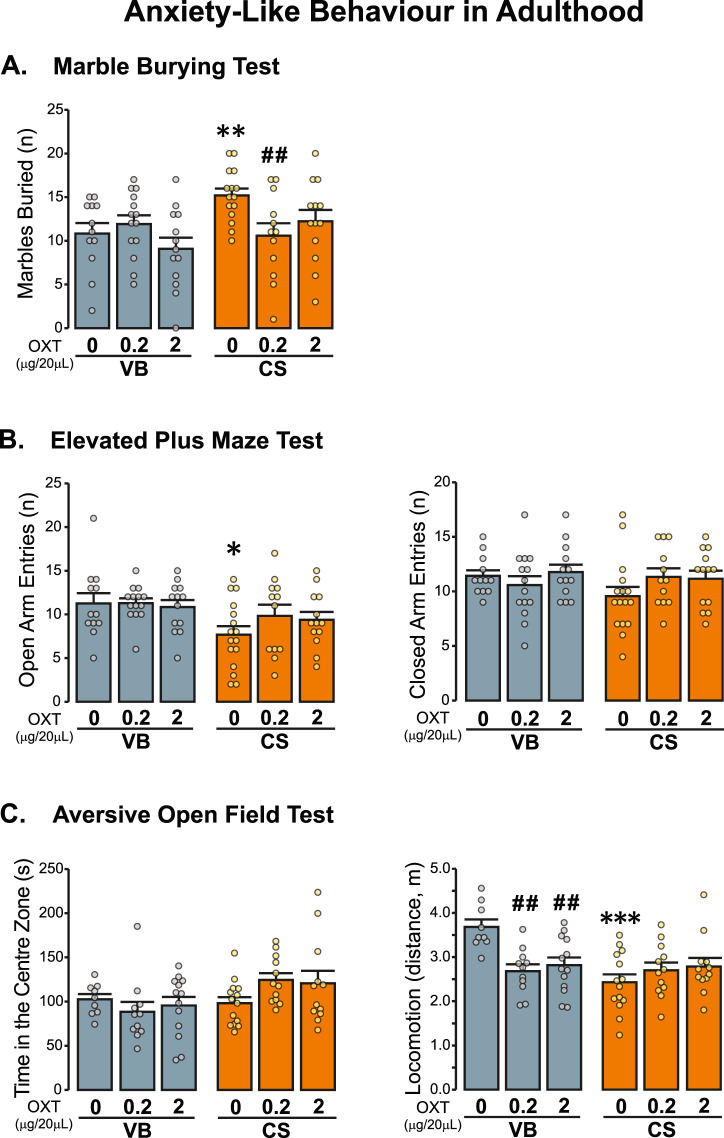
Effects of postnatal OXT administration on C-section-associated anxiety phenotype. **A***Early postnatal treatment with low-dose OXT decreased the number of marbles buried in the CS group*. Mode of delivery effect (*F* (1, 73) = 4.716, *p* = 0.033); treatment effect (*F* (2, 73) = 2.258, *p* = 0.112); mode of delivery × treatment (*F* (2,73) = 3.351, *p* = 0.041) ([VB control *n* = 12, VB 0.2 OT *n* = 14, VB 2 OT control *n* = 13, CS control *n* = 15, CS 0.2 OXT *n* = 12, CS 2 OXT *n* = 13]). **B** *OXT administration did not improve C-section-induced anxiety-like behaviour in the elevated plus maze.* Mode of delivery effect (*F* (1, 74) = 7.614, *p* = 0.007); treatment effect (*F* (2, 74) = 0.663, *p* = 0.518); mode of delivery × treatment (*F* (2,74) = 0.820, *p* = 0.444). **C** There were no significant differences in the number of entrances of the closed arms of the EPM. Mode of delivery effect (*F* (1, 74) = 0.843, *p* = 0.361); treatment effect (*F* (2, 74) = 0.829, *p* = 0.441); mode of delivery × treatment (*F* (2,74) = 1.497, *p* = 0.231). ([VB control *n* = 12, VB 0.2 OT *n* = 14, VB 2 OT control *n* = 13, CS control *n* = 16, CS 0.2 OXT *n* = 12, CS 2 OXT *n* = 13]).**C** *There were no significant effects on time spent in central zone of an aversive-open field. Early postnatal treatment did not reverse C-section-mediated effects on the total distance travelled in an aversive open-field arena.* Time in the central zone: Mode of delivery effect (*F* (1, 65) = 8.167, *p* = 0.006); treatment effect (*F* (2, 65) = 2.079, *p* = 0.133); mode of delivery × treatment (*F* (2,65) = 7.848, <0.001) ([VB control *n* = 9 VB 0.2 OT *n* = 11, VB 2 OT control *n* = 13, CS control *n* = 14, CS 0.2 OXT *n* = 12, CS 2 OXT *n* = 12]). Total distance travelled: Mode of delivery effect (*F* (1, 65) = 5.531, *p* = 0.022); treatment effect (*F* (2, 65) = 0.713, *p* = 0.261); mode of delivery × treatment (*F* (2,65) = 2.164, *p* = 0.123) ([VB control *n* = 9 VB 0.2 OT *n* = 11, VB 2 OT control *n* = 13, CS control *n* = 14, CS 0.2 OXT *n* = 12, CS 2 OXT *n* = 12]). **A**–**C** Two-way ANOVA, followed by LSD post-hoc. Data are represented as Mean ± Standard Error of the Mean (S.E.M.). Male offspring in each group is derived from three independent litters. *p < 0.05, *p < 0.01 and ***p < 0.001. OXT oxytocin, VB vaginal birth, CS C-section.

To extend our investigation on the effects of the early-life pharmacological manipulation of OXT system on the C-section-associated behavioural phenotype, adult male VB and CS offspring were tested in a variety of anxiety-like-behavioural paradigms (Fig. 3). In adulthood, as expected, CS showed increased anxiety-like behaviour as they buried significantly more marbles in comparison to VB in the marble burying test (Fig. 3a). Interestingly, postnatal injection with the low-dose OXT attenuated these effects (Fig. 3a). In addition, we measured anxiety-like behaviour in the EPM (Fig. 3b). Although we found that C-section reduced the number of entrances into the open arms of the EPM in comparison to the control group, there were no significant effects of OXT treatment in this test (Fig. 3b). When mice were tested in the aversive open field test, we saw no impact of either mode of delivery or OXT treatment on the time spent in the centre zone of the arena (Fig. 3c). C-section reduced the total distance travelled in the open field with no effects of OXT on ameliorating these effects (Fig. 3c). Interestingly, treatment with OXT significantly reduced the distance travelled in the VB, but not the CS group (Fig. 3c). Given that anxiety and depression are highly comorbid pathologies in humans [55], we tested whether mice born by C-section exhibit depressive-like behaviour using the forced-swim test, as well as, the potential for early-life treatment with OXT to exert anti-depressive response in mice. However, no significant alterations were observed across the groups (Fig. S4).

### Early-life OXT modulated immune response in adult mice

**Fig. 4:**
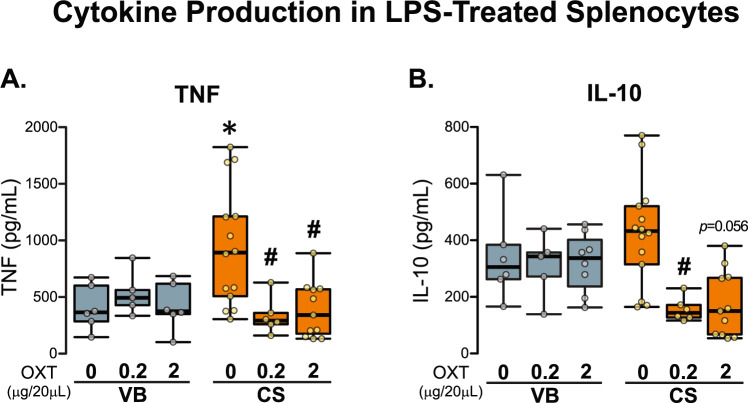
Early-life OXT administration induced long-lasting effects on immune function. **A***Early-life OXT attenuates TNF-α secretion in CS splenocytes stimulated with LPS*. Mode of delivery effect (*x*^2^ = 0.432; df = 1; *p* = 0.511) and treatment effect (*x*^2^ = 7.994; df = 2; *p* = 0.018) ([VB control *n* = 6, VB 0.2 OT *n* = 5, VB 2 OT control *n* = 6, CS control *n* = 14, CS 0.2 OXT *n* = 6, CS 2 OXT *n* = 11]). **B** *Early-life OXT decreases IL-10 secretion in CS splenocytes stimulated with LPS in CS group.* Mode of delivery effect (*x*^2^ = 227.000; df = 1; *p* = 0.177) and treatment effect (*x*^2^ = 11.805; df = 2; *p* = 0.003) ([VB control *n* = 6, VB 0.2 OT *n* = 5, VB 2 OT control *n* = 8, CS control *n* = 14, CS 0.2 OXT *n* = 6, CS 2 OXT *n* = 11]). Kruskal–Wallis test followed by Mann–Whitney. Male offspring in each group is derived from three independent litters. Data are represented as Median ± Interquartile range. *p < 0.05 for mode of delivery effect and ^#^*p* < 0.05 for treatment effect within the group. OXT oxytocin, VB vaginal birth, CS C-section.

To combine the pharmacological evidence of a role of OXT in ameliorating specific C-section-associated behavioural phenotype with the established link between OXT and the neuro-immune axis [56], we assessed whether early-life treatment with OXT could modulate the immune response in adult male CS offspring. Interestingly, when splenocytes from CS mice were challenged with LPS they produced significantly more TNFα than the splenocytes from VB mice (Fig. 4a). This aberrant immune response was attenuated by administration of low-dose and high-dose OXT early in life (Fig. 4a). Moreover, treatment with low-dose and high-dose OXT reduced IL-10 production in CS splenocytes (Fig. 4b), despite not affecting IL-4 and IL-6 levels (Table 1).

**Table 1:** There were no significant effects of C-section or OXT on splenocytes IL-4 and IL-6 secretion after LPS stimulation.

Cytokines	VB			CS		
	*0 OXT (µg/20* *µL)*	*0.2 OXT (µg/20* *µL)*	*2 OXT (µg/20* *µL)*	*0 OXT (µg/20* *µL)*	*0.2 OXT (µg/20* *µL)*	*2 OXT (µg/20* *µL)*
*Il-4*	0.64 ± 0.06	0.61 ± 0.09	0.55 ± 0.11	0.65 ± 0.10	1.17 ± 0.29	0.64 ± 0.06
*Il-6*	491.0 ± 57.64	700.8 ± 284.20	531.3 ± 81.48	854.6 ± 136.50	746.3 ± 313.90	372.7 ± 86.00

Data are represented as mean ± S.E.M.

*OXT* oxytocin, *VB* vaginal birth, *CS* C-section, *LPS* lipopolysaccharide.

### Early-life postnatal OXT improved intestinal motility deficits in adult C-section offspring

Birth by C-section is known to interfere with the wiring of the gut-brain axis in mice [9]. To further characterise whether birth by C-section could affect gastrointestinal function in male offspring and whether the pharmacological treatment with OXT could have beneficial effects, we used the carmine red assay as an in vivo marker of intestinal transit. Here we show that CS mice have faster gastrointestinal transit when compared with VB mice. Very little is known about the developmental effect of OXT administration on gut physiology and here we show that treatment with low-dose OXT early in life reversed these effects (Fig. S5a). To test whether this effect of OXT on gastrointestinal motility correlates with changes in gastrointestinal permeability, we measured transepithelial flux (from the gut lumen to the circulating blood) of 4 kDa FITC- dextran in the VB and CS offspring. However, no significant alterations in macromolecular permeability of the GI tract were observed across the groups (Fig. S5b).

## Discussion

Recent research has established that birth by C-section can result in different pattern of behaviour in humans [6–8] and in animals [9–11]. Recent discoveries point to a pivotal role for OXT in regulating general well-being through its influence on neuro-immune-endocrine pathways [57]. Here we demonstrate that pharmacological manipulation of the OXT system during the early postnatal period (P1-P5) improves social and anxiety-like behaviours, as well as the immune response later in life in the C-section mice model. OXT levels are demonstrated to be increased in vaginally born, but not in C-section neonates [58].The results obtained from this study demonstrate that there is an developmental window sensitive to manipulations of the OXT system, hormone replacement with OXT in early-life which may be capable of preventing some of the lifelong behavioural and physiological impairments induced by the mode of delivery.

OXT is fundamental for establishing maternal-offspring attachment and social behaviour in mammals [12–15]. In our studies the most consistent finding in relation to behavioural changes is that postnatal treatment with OXT reversed C-section-mediated social recognition deficits. At P10, CS offspring spent less time in the maternal nest which was completely restored by the high dose of OXT. The C-section-associated attachment deficits were not associated with differences in maternal care received by the foster dam. Nonetheless, both doses of OXT applied to the offspring increased maternal care in all groups. This is important as OXT is known for its role on strengthening maternal-offspring bonds [59, 60]. Although our maternal care analysis was limited to a short timeframe, differences in maternal care may also underlie some of the long-lasting effects we observe in our treatment with OXT. A more comprehensive analysis of maternal behaviour is warranted to understand the role of early-life OXT treatment and subsequent behaviour development outcomes. Early orientation to specific odour cues is critical to mammalian offspring survival and to social behaviour developmental trajectories [61]. As expected, adult CS offspring showed deficits in social novelty preference which was fully restored by low-dose OXT treatment early-in-life. Our results are in line with other studies that demonstrate that early life administration of OXT can reverse long-lasting social behavioural deficits in the contactin-associated protein-like 2 (*CNTNAP2*) model of ASD [62]. Moreover, OXT treatment after birth prevents social and cognitive deficits OXT system function in adult mice deficient for MAGE Family Member L2 (*Magel2)* gene, which is involved in Prader–Willi Syndrome and ASD [45].

As previously shown, CS offspring exhibited enduring deficits in anxiety-like behaviour in the marble burying test, elevated-plus maze and in the aversive open-field tests. In addition to its role in social behaviour, OXT has been associated with anxiolytic and anti-stress effects in the brain [17]. Here we demonstrated that postnatal treatment with a low dose of OXT reduced anxiety in the marble burying test, but not in the elevated-plus maze or in the aversive open-field in male CS offspring. Although these three behavioural models evaluate the tendency of mice to engage in exploratory behaviour in an aversive space [63], the marble burying is also widely thought as a measure of repetitive behaviour [64], which may be also be relevant to ASD.

Here we investigated the effects of the delivery mode on peripheral levels of OXT (in plasma) as well as *Oxtr* and *Avpr1a* mRNA expression in the PVN in male adult offspring. Although mode of delivery did not affect plasma OXT, postnatal pharmacological manipulation of the OXT system increased the peptide plasma levels in adulthood in both VB and CS groups. In a different study, using a similar dosage and treatment protocol, Meziane et al. (2014) [45] demonstrated that a single dose of subcutaneous OXT administered to neonates can bypass the blood-brain barrier and act in the brain using matrix-assisted laser desorption/ionisation. Surprisingly, *Oxtr* and *Avpr1a* mRNA expression in the PVN were not changed in adulthood. Suggesting that alterations in OXT levels may depend more on post-transcriptional events and less on gene expression during this stage of life. Further, the potential changes to *Oxtr* expression in other brain regions that regulate social and anxiety-like behaviours warrant further investigation. Interestingly, with regards to behaviour the higher dose of OXT was more effective in preventing the CS-mediated effects in early postnatal days whereas the low dose of OXT was associated with long-term changes. These findings may indicate plasticity of the OXT system across the development. A similar dose response curve has been reported in several studies involving OXT and other peptides [40, 65]. A better understanding of the dynamic role of the OXT system during development would be required for the interpretation of these results [39, 66, 67]. Further, OXT developmental effects are not limited to the OXT system and behaviour and are suggested to have an organisational effect wiring the HPA-axis [68], the immune system [56], neurotransmitter systems such as GABAergic [35], noradrenergic and serotoninergic systems.

One of the main findings of this study is that early-life treatment with both doses of OXT prevented adult male CS offspring from immune hyper-reactivity and decreased the production of pro- (TNFα) and anti-inflammatory IL-10 cytokine production in splenocytes stimulated with LPS. Alterations in in immune system development has been previously associated with birth by C-section [69]. On the other hand, OXT has emerged as key factor regulating immune homeostasis through several different neuro-immune-endocrine networks [56, 70]. In addition, OXT is known to be involved in immune system maturation and by reinforcing immunotolerance by activation of a subset of CD4 + Foxp3 + CD25 + regulatory T cells (Tregs) [71]. Another noteworthy aspect is that OXT can modulate immune system maturation by activation of OXTR and AVPR present in the PVN [56] and in immune organs such as the thymus and spleen [72]. However, mRNA expression of *Oxtr* and *Avpr1a* in the PVN were not affected in the C-section model suggesting that this may not be the mechanism of immune dysfunction at play here. Further, the initial microbial acquisition due to C-section has been previously associated with abnormal immune responses in humans [73] and in mouse models [74]. Although we cannot rule out that the injection stress may affect both the HPA axis and immune system, the connection between microbiota and neuro-immune responses following birth by C-section requires further investigation [75].

Recently, the gut microbiota was shown to modulate the OXT system and improve social behaviour in animal models of ASD [76–78]. Thus, the relative contribution of such OXT-microbiota-immune interactions to the behavioural effects observed need to be explored in future studies. In the current study, we also observed an interesting physiological effect of postnatal low-dose OXT on restoring gastrointestinal transit in male CS adult mice without compromising gut permeability. In a previous study OXT was demonstrated to slow gastrointestinal motility, protect the intestinal barrier from inflammation and decrease macromolecular permeability [79]. Although speculative, the restoration of the gastrointestinal transit paralleled to restoring the immune system response could reflect an indirect developmental effect of OXT in the gut-brain axis network.

There is a growing interest in manipulation of the OXT system for potential therapeutic use. Our study opens an avenue for the investigation of how manipulation of the OXT system influences the wiring of the brain following C-section delivery. Although the translation of findings in animal models are limited, OXT is present in human breast milk [80] and breast-feeding initiation may also be adversely affected by C-section [81]. Some additional limitations to the study should be noted: here we focused on male mouse behaviour in adulthood as a direct follow-up to our previous findings in male mice [9] . As sex is an important biological variable for behaviour development and the OXT system [66, 82, 83], future studies should investigate the effects of C-section and OXT treatment on behaviour in females. In our previous study [9] we controlled for the effects of fostering by adding a cross-fostered group and demonstrated that cross-fostering has minimal effects on behaviour. However, for statistical, logistical, and ethical reasons we did not include extra groups in the current study. In addition, to minimise animal usage, all the behavioural tests were done in the same animals and we cannot rule out the possible confounding carryover effects from previous tests. Most of our findings on groups treated with OXT remained significant after controlling for litter effects (Supplementary Table 1), albeit some tests of anxiety and maternal attachment behaviour did not reveal differences between CS and VB groups after controlling for environment (Supplementary Table 1). It may be that these measures are more sensitive to litter or handling effects during early-life that may be masking differences between the groups [9, 40, 84].

Finally, as a rise in unplanned C-section deliveries are a global trend [85] the data from this study suggest that targeting OXT may serve as a novel and worthy target for improving health outcomes associated with the procedure.

## Funding and disclosure

This publication has emanated from research supported in part by a Centre grant from Science Foundation Ireland (SFI) to the APC Microbiome Institute under Grant Number SFI/12/RC/2273_P2. It was funded from the European Community’s Seventh Framework Programme Grant MyNewGut under Grant Agreement No. FP7/2007–2013 and Dept Agriculture, Food & the Marine, Ireland funded TODDLERFOOD: Food Solutions for Replenishing Disrupted Microbiota in Toddlers (2014–2018) and SMARTFOOD**:** Science Based ‘Intelligent’/Functional and Medical Foods for Optimum Brain Health, Targeting Depression and Cognition (2013–2017). LHM was funded by Science Without Borders, Coordenação de Aperfeiçoamento de Pessoal de Nível Superior (CAPES), under the Grant Agreement Number No. 11601-13-2. The authors have nothing to disclose. Open Access funding provided by the IReL Consortium.

## Supplementary information

Supplemental material
