# Supplementary material for: Early-life oxytocin attenuates the social deficits induced by caesarean-section delivery in the mouse
Source: Neuropsychopharmacology. 2021 May 26;46(11):1958–68. doi: 10.1038/s41386-021-01040-3 (PMC8429532; doi:10.1038/s41386-021-01040-3)
Supplement: Supplementary file 1 — Supplemental material [file 41386_2021_1040_MOESM1_ESM.pdf]

Supplementary Figures

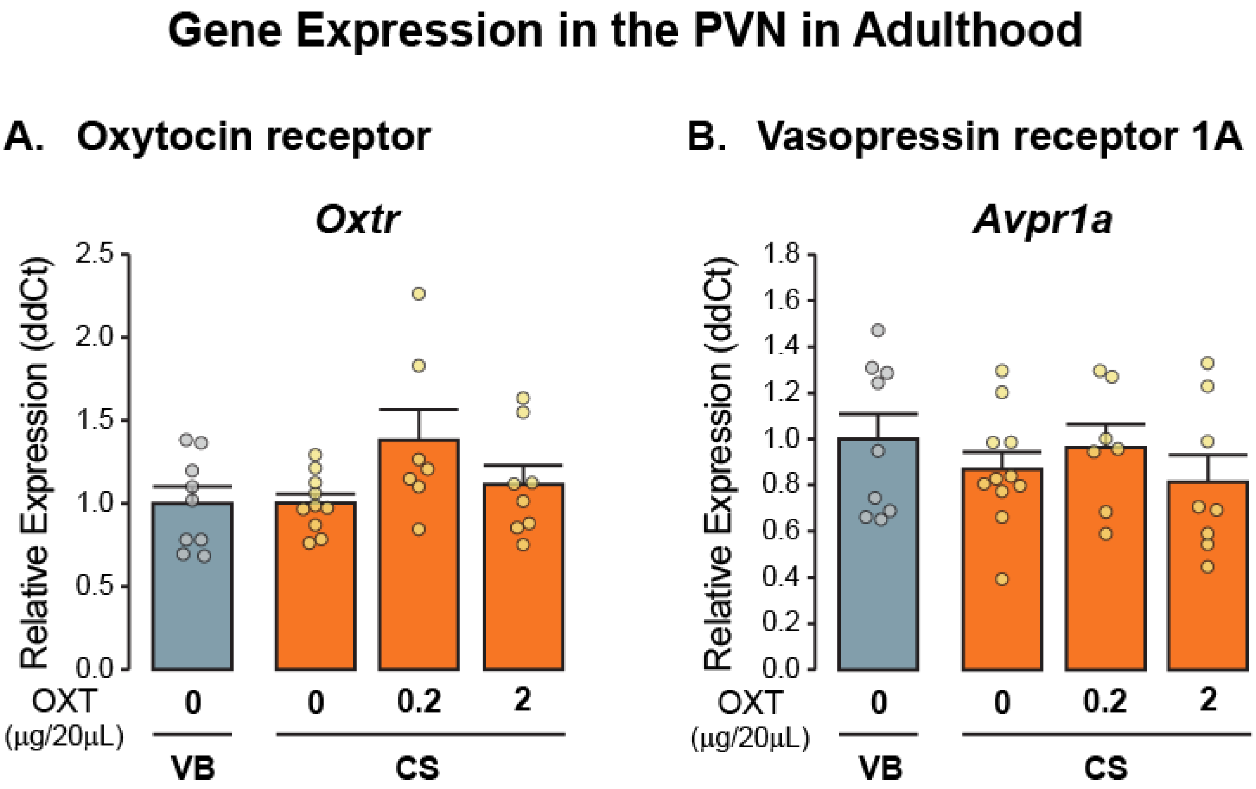

Figure S1. OXT and AVP1a receptors mRNA expression in the paraventricular nucleus of hypothalamus (PVN). A) There were no significant effects on OXT receptor mRNA expression in the PVN. *VB x CS* ***(****t=0.622; df=18; p=0.542). CS x CS + treatment (F (2,23) =0.850, p=0.440). ([VB control n= 9, CS control n=11, CS 0.2 OXT n=7, CS 2 OXT n=8]).* ***B) There were no significant effects on AVP1a receptor mRNA expression in the PVN.*** *(t=1.018; df=18; p=0.322), CS x CS + treatment (F (2,23) = 0.531, p=0.595). ([VB control n= 9, CS control n=11, CS 0.2 OXT n=7, CS 2 OXT n=8]).* ***A-B)*** *Student's t -test and One-way ANOVA. Data are represented as Mean ± Standard Error of the Mean (S.E.M.) Male offspring in each group derived from three independent litters. *p<0.05 for mode of delivery effect. #p< 0.05 for treatment effect within the group. OXT, oxytocin; VB, vaginal birth; CS, C-section.*

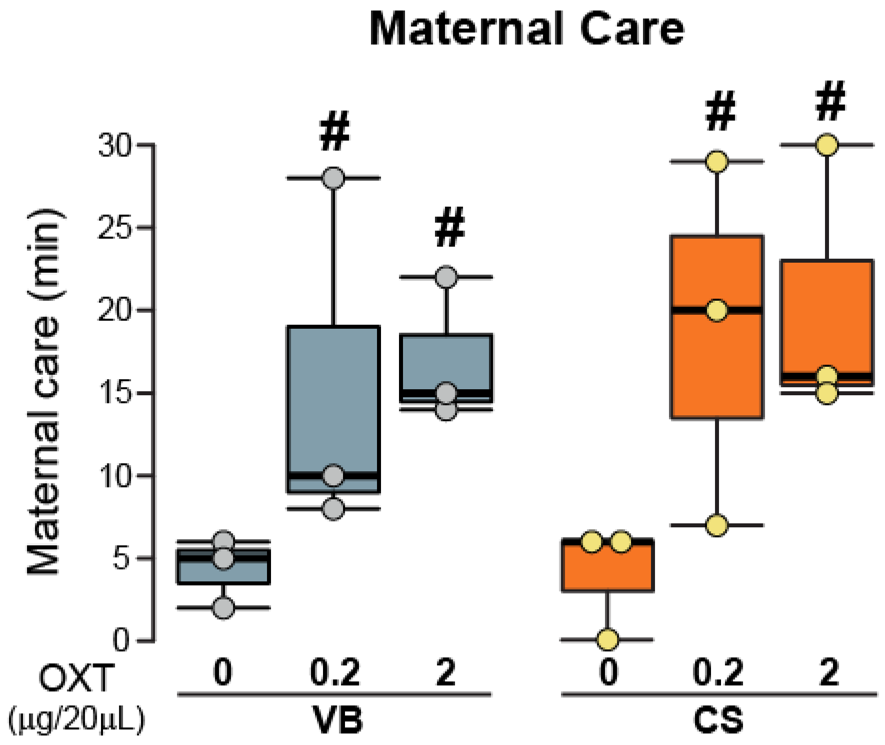

Figure S2. Mode of delivery did not affect time spent on maternal care behaviours. *To exclude the possibility that differences in offspring phenotype investigated in this study are due to variation in maternal care per se, we assessed maternal care behaviour on P6. There was no significant effect of the mode of delivery (x^2= 0.282; df=1; p=0.595). However, when pups were treated with OXT there was a significant increase on the time the mother spent engaged in high care behaviours across all the groups (x^2= 11.615; df=2; p=0.003). n=3 dams per group. Kruskal-Wallis test followed by Mann-Whitney U test. Data are represented as Median ± Interquartile range. $^{\#}p < 0.05$ for treatment effect within the group. OXT, oxytocin; VB, vaginal birth; CS, C-section.*

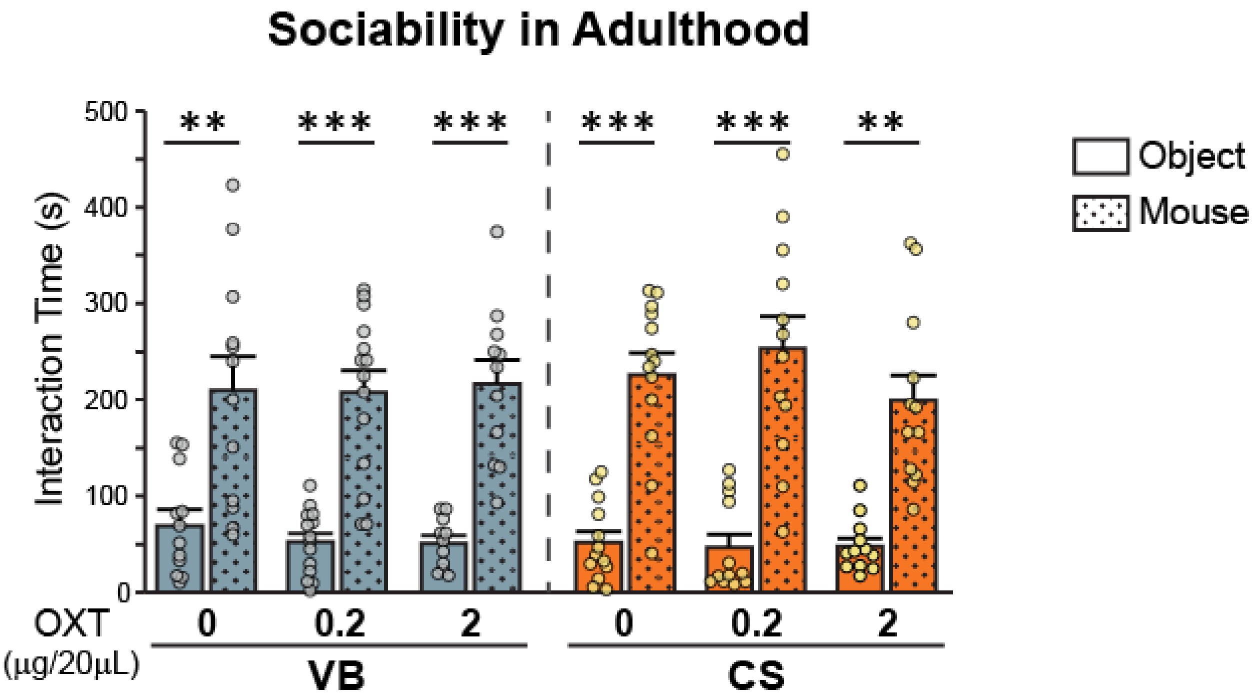

Figure S3. There were no significant differences on sociability across all groups in sociability. *VB control t (11) = 3.893, p=0.003, n=12; VB 0.2 OXT t (13) =6.140, p<0.0001, n=14; VB 2 OXT t (11) =6.107, p<0.0001, n=12; CS control t (12) = 6.495, p=0.0001, n=13; CS 0.2 OXT t (11) = 5.395, p<0.0.001, n=12; CS 2 OXT t (11) = 5.118, p<0.0001, n=12. Paired Student's t test comparing interaction time with mouse to an object. Data are represented as Mean ± Standard Error of the Mean (S.E.M.). Male ofoffspring in each group derived from three independent litters.*

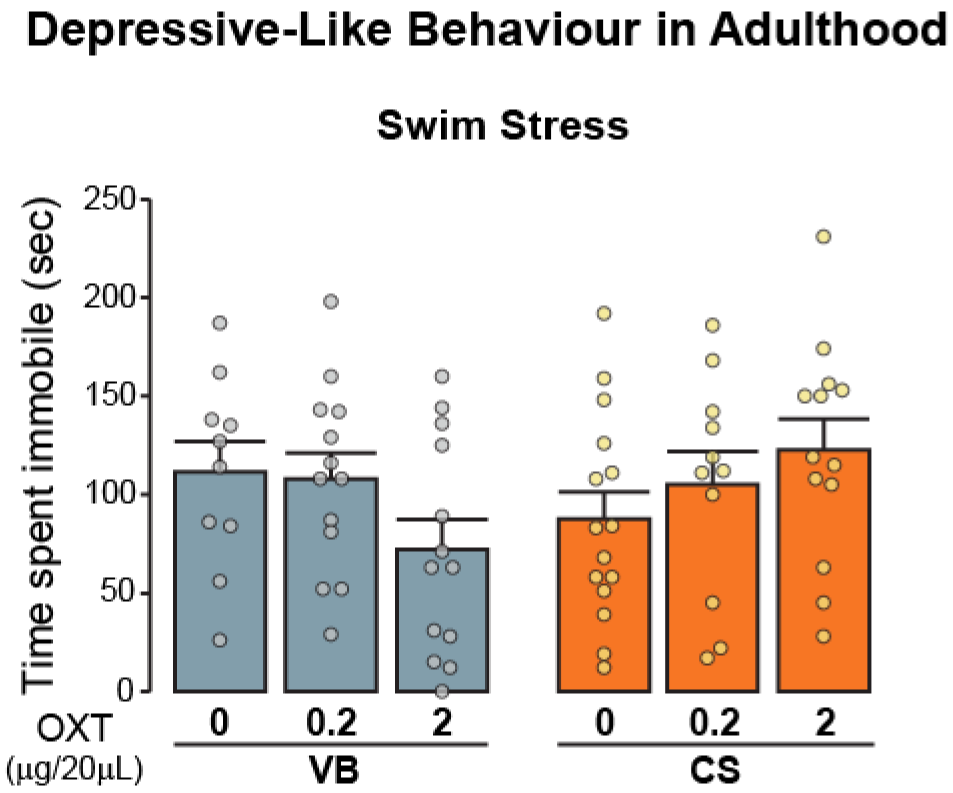

Figure S4. There were no significant differences in the forced swim test. *Mode of delivery effect (F (1, 74) = 0.881, p=0.351); treatment effect (F (2, 74) = 0.295, p= 0.745); mode of delivery x treatment (F (2,74) = 2.300, p=0.107). ([VB control n= 12, VB 0.2 OT n=14, VB 2 OT control n=13, CS control n=16, CS 0.2 OXT n=12, CS 2 OXT n=13]). Two-way ANOVA, followed by LSD post-hoc. Data are represented as Mean ± Standard Error of the Mean (S.E.M.). Male ofoffspring in each group derived from three independent litters. ***p<0.0001 for mode of delivery effect. $^{\#\#}p< 0.01$ for treatment effect within the group. OXT, oxytocin; VB, vaginal birth; CS, C-section.*

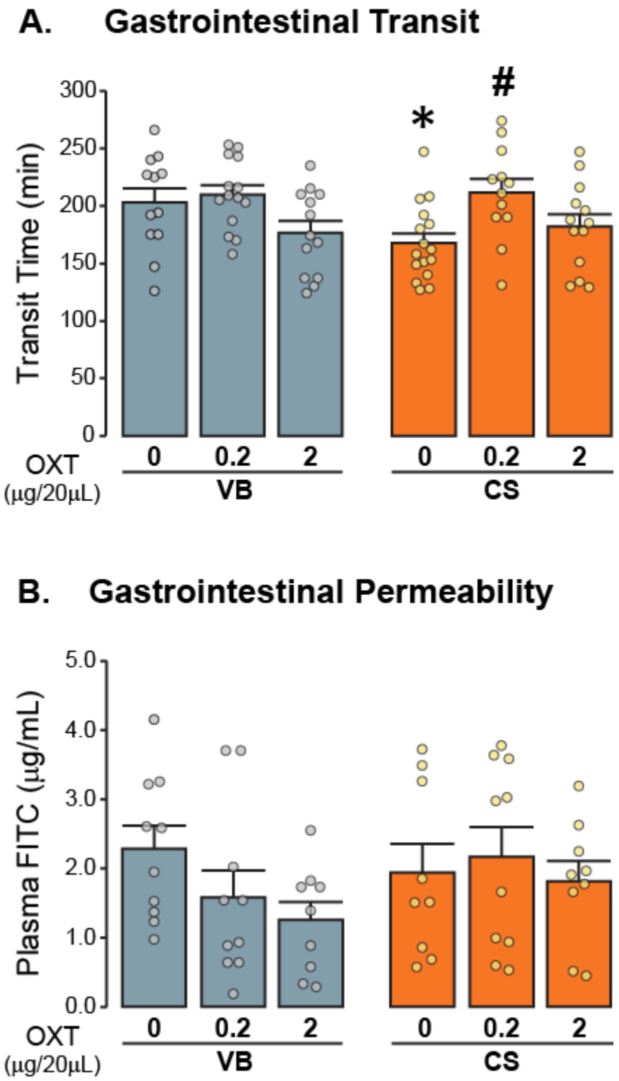

Figure S5. A) OXT effects on gastrointestinal motility and permeability in C-section. A) CS exhibited faster intestinal transit in comparison to VB, which was fully reversed by the low-dose OXT treatment in early-life. *Mode of delivery effect (F (1, 74) = 1.278, p=0.262); treatment effect (F (2, 74) = 5.238, p= 0.007); mode of delivery x treatment (F (2, 74) = 2.523, p=0.087). ([VB control n= 12, VB 0.2 OT n=14, VB 2 OT control n=13, CS control n=16, CS 0.2 OXT n=12, CS 2 OXT n=13]).* ***B) Effects of early-life treatment with OXT to gastrointestinal permeability****. There was no significant effect of mode of delivery or OXT treatment in gastrointestinal permeability as measured by plasma Fluorescein isothiocyanate (FITC)-dextran concentration (µg/mL). Mode of delivery effect (F (1, 74) = 3.449, p=0.068); treatment effect (F (2, 74) = 0.178, p= 1.774); mode of delivery x treatment (F (1, 74) = 0.3291, p=0.721). ([VB control n= 12, VB 0.2 OT n=10, VB 2 OT n=10, CS control n=14, CS 0.2 OXT n=11, CS 2 OXT n=10]).* ***A-B)*** *Two-way ANOVA, followed by LSD post-hoc. Data are represented as Mean ± Standard Error of the Mean (S.E.M.). Offspring in each group derived from three independent litters. Two-way ANOVA, followed by LSD post-hoc. *p<0.05 for mode of delivery effect.* $^{\#}p < 0.05$ *for treatment effect within the group. OXT, oxytocin; VB, vaginal birth; CS, C-section.*

Supplementary Table 1.

	Mixed-effects ANOVA				Multi-comparisons test
**Experiment**	**NumDF**	**DenDF**	**F value**	**Pr(>F)**	
Pups- Ultrasonic vocalization (# calls)	5	195	12.402	**1.87E-10**	p<0.001 VB + 0 OXT *vs* VB+0.2 OXT; p<0.001 CS + 0 OXT vs CS+0.2 OXT
Pups- Maternal attachment (maternal preference)	5	124.48	1.4984	0.1951	N/A
# Marbles buried	5	79	3.6984	**0.00465**	p=0.06 VB+ 0 OXT *vs* CS+ 0 OXT; p<0.05 CS + 0 OXT vs CS+0.2 OXT
Sociability -Investigation time (mouse)	5	72.662	0.5383	0.7466	N/A
Sociability -Investigation time (object)	5	75	0.5077	0.7695	N/A
Social recognition Investigation time (novel mouse)	5	66.567	2.4736	**0.0408**	ns
Social recognition Investigation time (familiar mouse)	5	66.952	3.3174	**0.00977**	p<0.01 VB + 0 OXT vs CS+ 0 OXT; p<0.05 CS + 0 OXT vs CS+0.2 OXT
Elevated plus maze- Entrances (open-arms)	5	80	2.5313	**0.03526**	p=0.07 VB + 0 OXT vs CS+ 0 OXT
Elevated plus maze- Entrances (closed-arms)	5	77.067	1.3428	0.2554	N/A
FITC	5	57	1.2486	0.2988	N/A
Forced swim test- Immobility time	5	75	1.6933	0.1466	N/A
Transit time	5	77.151	5.0707	**0.00045**	p<0.05 VB+ 0 OXT *vs* CS+ 0 OXT; p<0.01 CS + 0 OXT vs CS+0.2 OXT
Plasma oxytocin concentration	5	44.996	3.6692	**0.0072**	p<0.01 CS + 0 OXT vs CS+ 2 OXT
Aversive open-field (time in the centre)	5	71	2.3979	**0.04561**	ns
Aversive open-field (total distance)	5	71	5.3685	**0.0003**	p<0.001 VB+ 0 OXT *vs* CS+ 0 OXT; p<0.001 VB+ 0 OXT vs VB+ 0.2 OXT; VB+ 0 OXT vs VB+ 2 OXT
Splenocytes stimulation- IL10 concentration	5	51.875	4.0863	**0.00334**	p<0.001 CS + 0 OXT vs CS+0.2 OXT
Splenocytes stimulation-TNFα concentration	5	49	6.0932	**0.00018**	p<0.001 VB+ 0 OXT *vs* CS+ 0 OXT; p<0.01 CS + 0 OXT vs CS+0.2 OXT; p<0.01 CS + 0 OXT vs CS+2 OXT
Splenocytes stimulation-IL-4 concentration	5	58	0.8256	0.5366	N/A
Splenocytes stimulation-IL-6 concentration	5	58	2.2287	0.06344	N/A
qRT-PCR -Oxtr (relative expression)	3	31.279	2.8102	0.05554	N/A
qRT-PCR -Avpr1a (relative expression)	3	33.403	0.686	0.5669	N/A

Litter and cage effects. The table compares litter and cage effects for behavioural data in adult mice. Data were analysed with mixed-effects ANOVA followed by multi-comparisons between groups VB, vaginal birth; CS, C-section; OXT, oxytocin; TNF, tumor necrose factor; IL, interleukin.
